# Quadruple‐refocused spin‐locking: A robust method for high‐amplitude T_1ρ_ imaging

**DOI:** 10.1002/mrm.30621

**Published:** 2025-06-28

**Authors:** Cai Wan, Maximilian Gram, Wei He, Zheng Xu, Qingping Chen, Sebastian Littin, Thomas Lange, Maxim Zaitsev

**Affiliations:** ^1^ School of Electrical Engineering Chongqing University Chongqing China; ^2^ Division of Medical Physics, Department of Diagnostic and Interventional Radiology, University Medical Center Freiburg, Faculty of Medicine University of Freiburg Freiburg Germany; ^3^ School of Pharmacy and Bioengineering Chongqing University of Technology Chongqing China; ^4^ Department of Internal Medicine I University Hospital Würzburg Würzburg Germany; ^5^ Experimental Physics 5 University of Würzburg Würzburg Germany

**Keywords:** artifacts compensation, field inhomogeneity, quantitative MRI, spin locking, T_1ρ_ relaxation

## Abstract

**Purpose:**

Longitudinal relaxation time in the rotating frame (T_1ρ_) is a source of tissue‐specific contrasts, with applications in detecting myocardial fibrosis, liver fibrosis, and early‐stage osteoarthritis. However, T_1ρ_ measurements are sensitive to static (B_0_) and radiofrequency (B_1_) magnetic field inhomogeneities. Improving the accuracy of T_1ρ_ quantification can enable earlier and more reliable detection of pathological changes, providing a basis for early intervention. Therefore, we have developed an improved, quadruple‐refocused spin‐locking (QR‐SL) technique based on existing preparation schemes to obtain a more robust compensation for B_0_ and B_1_ field inhomogeneities.

**Methods:**

The QR‐SL module consists of four 180° refocusing pulses with opposite phases and five spin‐locking (SL) pulses with phase cycling according to the rotary‐echo principle. The performance of the proposed QR‐SL module is evaluated through numerical simulations and experimental validation in comparison to composite‐SL (C‐SL), balanced‐SL (B‐SL), and triple‐refocused‐SL (TR‐SL) preparation modules.

**Results:**

Numerical simulations indicate that the QR‐SL module demonstrates improved tolerance to a range of B_0_ and B_1_ field inhomogeneities compared to the other three modules. In scenarios involving inhomogeneities of both fields, the experimental results show that the residual sum of squares of the QR‐SL module was decreased by 24.3%, 68.9%, and 12.5% for in vivo knee cartilage, respectively, compared to the composite SL, balanced SL, and triple‐refocused SL preparation modules.

**Conclusion:**

The QR‐SL module has the potential to produce more accurate T_1ρ_ maps, while minimizing artifacts. Consequently, the QR‐SL module is more favorable for T_1ρ_ quantification, especially for low‐field and ultralow‐field quantitative MRI.

## 1 INTRODUCTION

In magnetic resonance imaging (MRI), healthy and diseased tissues can be distinguished by differences in their relaxation times (T_1_ and T_2_), magnetization transfer, diffusion properties, or perfusion effects. As an additional potential source of contrast, spin–lattice relaxation time in the rotating frame, T_1ρ_, has been studied intensively. It was first described by Redfield^1^ in 1955 in nuclear magnetic resonance (NMR) and introduced to the MRI field by Sepponen et al.^2^

Decades after its initial development, T_1ρ_ mapping has become an emerging quantitative MRI technique. This is attributed to the sensitivity of the T_1ρ_ relaxation mechanism to low‐frequency dynamics at the molecular level. The relevant low‐frequency molecular dynamic processes in tissues involve the proton exchange among hydroxyl or amide groups found in proteins.^3^, ^4^ Notable in vivo applications of T_1ρ_ mapping include myocardial fibrosis,^5^, ^6^, ^7^ liver fibrosis,^8^, ^9^, ^10^ and early‐stage osteoarthritis.^11^, ^12^, ^13^

T_1ρ_‐weighted images are acquired by applying an on‐resonance continuous‐wave radiofrequency (RF) pulse in the transverse plane, called a spin‐locking (SL) pulse. The SL pulse is characterized by its long duration and relatively low energy. It effectively decelerates the relaxation process in the transverse plane by constraining the spin to rotate around the RF magnetic field, B_1_. Initially, the magnetization is tilted by 90° and then locked with the continuous RF pulse, effectively suppressing certain conventional transverse relaxation mechanisms. However, T_1ρ_ quantification is strongly affected by inhomogeneities of both the static magnetic field (B_0_) and the applied RF field (B_1_).^2^ Various methods have been proposed to compensate for the sensitivity of T_1ρ_ quantification to field inhomogeneities. Charagundla et al. used a rotary‐echo SL module to weaken the dependence of MR signal on variations in the B_1_ field.^14^ The original long‐duration SL pulse was divided into two equal‐duration pulses with opposite phases. Nevertheless, the SL pulse remained susceptible to inhomogeneity of the B_0_ field. Zeng et al. introduced a 180° pulse within a SL sequence to compensate for the B_0_ field inhomogeneity.^15^ However, this approach was unable to eliminate artifacts arising from flip‐angle imperfections. Witschey et al. proposed a composite‐SL (C‐SL) pulse that demonstrated insensitivity to variations in both B_0_ and B_1_ fields.^16^ The C‐SL module represents an enhancement of the ΔB_0_‐insensitive SL module initially proposed by Zeng et al., wherein the phase of the final 90° pulse has been modified to effectively reverse the magnetization. The C‐SL module still requires a precise 180° refocusing pulse to effectively counteract magnetic field variations. An alternative approach to mitigate the sensitivity of the SL pulse to field inhomogeneity, termed paired self‐compensated SL (PSC‐SL), was proposed by Mitrea et al.^17^ This SL pulse scheme is further divided into pairs of pulses with opposite phases on either side of the 180° refocusing pulse. Mitrea et al. showed that in the case of high SL frequencies (*f*_SL_) and significant field inhomogeneities, the PSC‐SL module exhibited reduced susceptibility to artifacts. Then, Gram et al. proposed a double‐refocused pulse sequence, known as the balanced‐SL (B‐SL) module,^18^ with an additional 180° reverse‐phase refocusing pulse to compensate for inhomogeneities of both fields. The authors concluded that the B‐SL module outperformed existing SL modules, as demonstrated through numerical simulations and experimental validation performed at 7 T using an agarose phantom. Recently, Pala et al. investigated a variety of SL pulse types. They found that among hard pulses, the C‐SL and triple‐refocused‐SL modules (TR‐SL)^19^ showed greater robustness to field inhomogeneities, as confirmed by both Bloch simulations and experimental measurements. However, the performance of the B‐SL module relative to that of the C‐SL module remains to be determined. The SL module has been studied extensively and has shown continuous improvement. Nevertheless, these methods remain unable to fully compensate for the artifacts induced by field inhomogeneity across various scenarios.^18^, ^19^, ^20^ Therefore, further investigation is essential to achieve spin locking with fewer artifacts and to expand its applicability.

In this study, we propose a novel quadruple‐refocused‐SL (QR‐SL) preparation module. This module contains four 180° refocusing and five SL pulses. The application of four 180° refocusing pulses effectively mitigates the impact of B_0_ field inhomogeneity, and an even number of 180° pulses helps to balance the refocusing issue caused by imperfect B_1_ field distribution and other issues affecting 180° pulses.^18^ Although previous research has proposed using adiabatic pulses combined with SL as an effective alternative,^21^, ^22^, ^23^, ^24^ this study focuses on on‐resonance hard‐pulse SL sequences. In this work, we perform numerical simulations and experimental validations with the C‐SL, B‐SL, TR‐SL, and QR‐SL modules for various inhomogeneities of B_0_ and B_1_ fields.

## 2 THEORY

**FIGURE 1:**
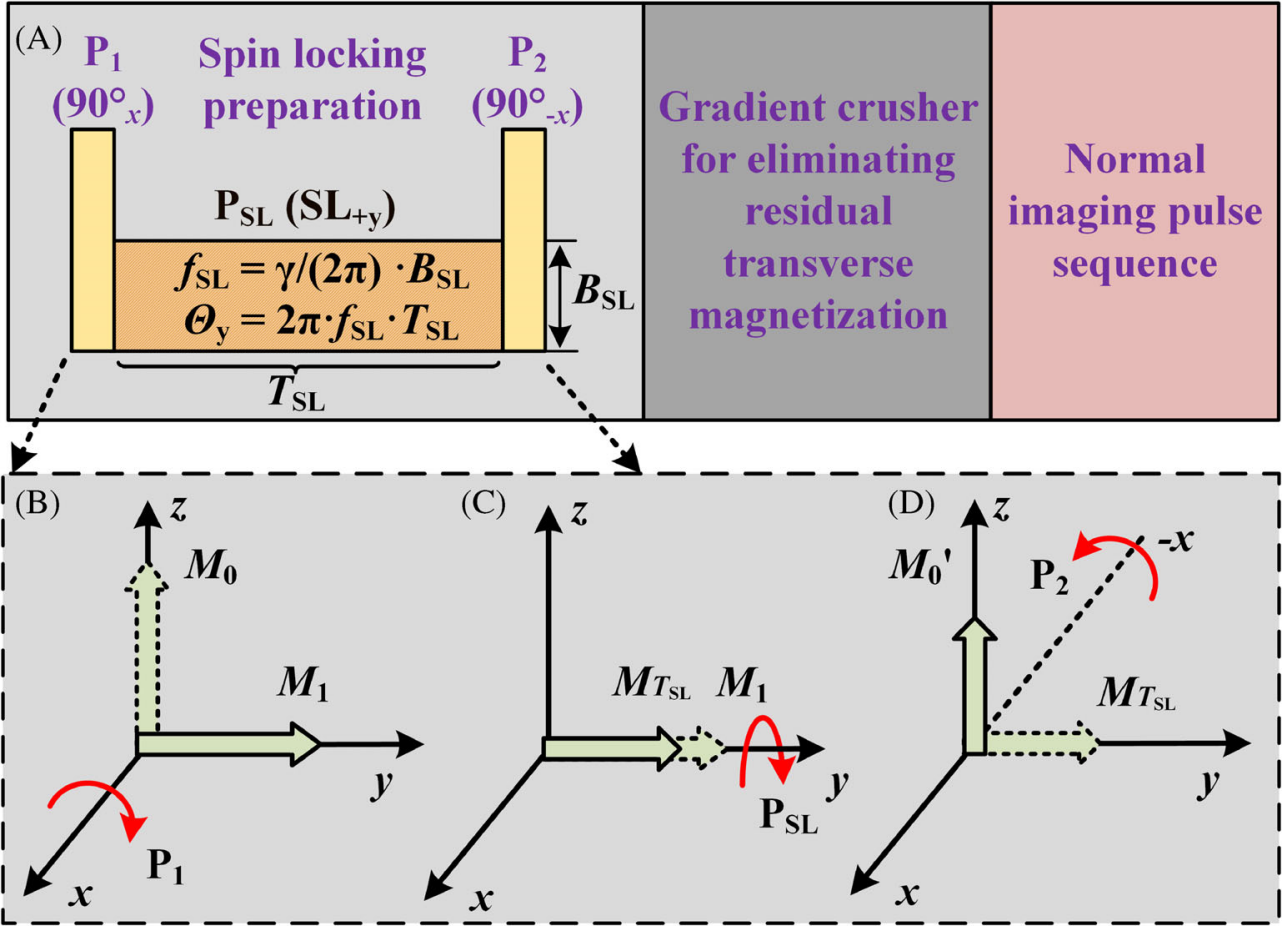
(A) Schematic representation of the typical spin‐locking (SL) module. (B) The initial 90° radio frequency pulse (P_1_) is applied along the x‐axis (rotating reference frame) to rotate the longitudinal magnetization, *M*_0_, into the transverse plane. (C) A spin‐locking pulse (P_SL_) along the y‐axis with a duration of *T*_SL_ is applied, rotating the magnetization by the total angle, *Θ*_*y*_, with the frequency, *f*_SL_. The symbol γ denotes the gyromagnetic ratio. (D) The concluding 90° RF pulse (P_2_) rotates the magnetization back to its original longitudinal direction.

The schematic diagram of a typical T_1ρ_ imaging sequence is shown in Figure 1. Initially, a 90° RF pulse is applied along the x‐axis (P_1_) to tilt the magnetization into the transverse plane (Figure 1B). Then, an SL pulse (P_SL_) is applied along the y‐axis with a duration of SL time (*T*_SL_) and amplitude *B*_SL_ (Figure 1C). The rotation angle, *Θ*_*y*_, during this pulse is defined by 2π·*f*_SL_·*T*_SL_, where *f*_SL_ = γ/(2π)·*B*_SL_. The symbol γ denotes the gyromagnetic ratio. Next, a second 90° pulse (P_2_) in the opposite direction to P_1_ (−*x*‐axis) is applied to flip the magnetization back to its original longitudinal direction after the T_1ρ_ preparation (Figure 1D). Subsequently, a strong gradient crusher is used to eliminate residual transverse magnetization. Finally, the crusher is followed by a typical imaging sequence (Figure 1A), such as spin‐echo or gradient‐recalled‐echo sequences. The magnetization vector in the direction of the SL pulse is assumed to decay mono‐exponentially during *T*_SL_ at a rate of 1/T_1ρ_. Variations in *T*_SL_ produce different T_1ρ_‐weighted images. By fitting such series of differently weighted images to a mono‐exponential function on a pixel‐by‐pixel basis, a T_1ρ_ map is generated.

(1) $$S\left(T_{\mathrm{SL}}\right)=S_{0}e^{-\frac{T_{\mathrm{SL}}}{T_{1\rho }}}.$$

where *S*(*T*_SL_) is the measured signal intensity of the image at a particular *T*_SL_, and *S*_0_ is the hypothetical signal intensity at *T*_SL_ = 0.

**FIGURE 2:**
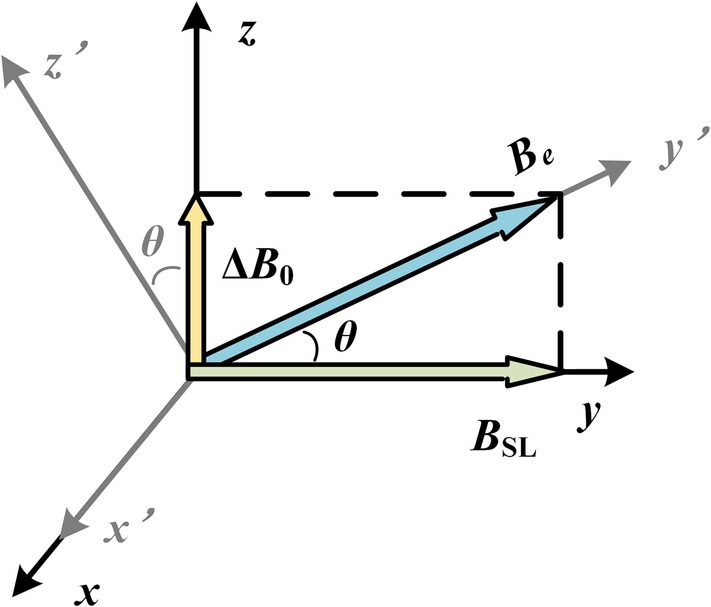
Effective spin‐locking (SL) strength and orientation changes in the rotating frame caused by the B_0_ field inhomogeneity. The x‐, y‐, and z‐axes denote the rotating frame of reference. ***B***_**e**_, effective magnetic field; ***B***_**SL**_, field strength of an SL pulse; **ΔB**_**0**_, reduced static field; *θ*, tilt angle.

However, in practice, the applied direction and amplitude of the SL pulse may be modulated due to inhomogeneities of the B_0_ and B_1_ fields of the MR scanner or the heterogeneity of the imaged object. This causes a deviation in the effective SL field direction and intensity from the nominal values, leading to a complex evolution of the magnetization instead of the desired mono‐exponential decay. The inhomogeneity of the B_1_ field leads to inaccuracies in the nominal 90° and 180° RF pulses and alters the frequency of the effective SL. The inhomogeneity of the B_0_ field results in a deviation of the direction of the effective SL field from the y‐axis by an angle *θ*, as shown in Figure 2. This intricate evolution of magnetization ultimately manifests as banding artifacts in the T_1ρ_‐weighted images and errors in T_1ρ_ quantification. As shown in Eq. (2) and Figure 2, a simple way to mitigate the effects of the B_0_ field inhomogeneity is to increase the SL field strength for *θ* to approach 0°. However, this would require a high B_1_ peak magnitude, which is limited by both the MR hardware performance and the specific absorption rate (SAR). Therefore, it is crucial to design preparation modules that are insensitive to the inhomogeneity of both B_0_ and B_1_ fields without substantially increasing the SL pulse magnitude. This can improve T_1ρ_‐weighted image quality and allow for accurate quantification of T_1ρ_ relaxation times.

(2) $$\theta =\tan^{-1}\left(\frac{\Delta \omega_{0}}{\omega_{\mathrm{SL}}}\right),$$

(3) $$\Delta \omega_{0}=2\pi \Delta f_{0},\omega_{\mathrm{SL}}=2\pi f_{\mathrm{SL}}=\gamma B_{\mathrm{SL}},$$

(4) $$\omega_{e}=\sqrt{\omega_{\mathrm{SL}}^{2}+\Delta \omega_{0}^{2}},$$

(5) $$B_{e}=B_{\mathrm{SL}}+\Delta B_{0}.$$

The design of SL pulses requires precise tracking of the magnetization evolution at each time point during the SL pulse, which can be effectively analyzed through the complete Bloch equation simulation. However, this process may become quite complex and computationally expensive. In the present work, we use an approach introduced in previous studies for the optimization of SL pulses based on matrix propagation.^16^, ^18^ Typically, the magnetization evolution can be effectively simulated by interleaving a series of rotation and relaxation matrices, which is sometimes referred to as the DANTE principle.^25^ The RF pulse action is represented by ***R***_***φ***_(*ψ*), where ***R*** denotes the rotation matrix, *φ* represents the phase of the RF pulse, and *ψ* indicates the rotation angle associated with the RF pulse. The magnetization evolution resulting from an RF pulse is generally expressed as ***M***_**t1**_ = ***R***_***φ***_(*ψ*)· ***M***_**t0**_, where ***M***_**t0**_ and ***M***_**t1**_ signify the magnetization vector before and after the pulse excitation, respectively. The rotation matrix expression for the rotation angle *ψ* of the magnetization around the x, y, and z axes in three‐dimensional space is given by

(6) $$\begin{array}{c} R_{x}\left(\psi \right)=\left(\begin{array}{ccc} 1 & 0 & 0\\ 0 & \cos\psi & \sin\psi \\ 0 & -\sin\psi & \cos\psi \end{array}\right);\\ R_{y}\left(\psi \right)=\left(\begin{array}{ccc} \cos\psi & 0 & -\sin\psi \\ 0 & 1 & 0\\ \sin\psi & 0 & \cos\psi \end{array}\right);\\ R_{z}\left(\psi \right)=\left(\begin{array}{ccc} \cos\psi & \sin\psi & 0\\ -\;\sin\psi & \cos\psi & 0\\ 0 & 0 & 1 \end{array}\right). \end{array}$$

Note that all these magnetization vectors follow clockwise rotation, and the coordinate system is left‐handed. Assuming that the SL pulse is applied along the y‐axis, spin relaxation matrix in the rotating frame under the action of an SL pulse of duration *T*_SL_ is denoted by

(7) $$E_{\rho }\left(T_{\mathrm{SL}}\right)=\left(\begin{array}{lll} e^{-T_{\mathrm{SL}}/T_{2\rho }} & 0 & 0\\ 0 & e^{-T_{\mathrm{SL}}/T_{1\rho }} & 0\\ 0 & 0 & e^{-T_{\mathrm{SL}}/T_{2\rho }} \end{array}\right).$$

where T_1ρ_ is the magnetization decay constant parallel to the SL pulse, and T_2ρ_ is the magnetization decay perpendicular to the SL pulse axis.

## 3 METHODS

### 3.1 SL preparation schemes

**FIGURE 3:**
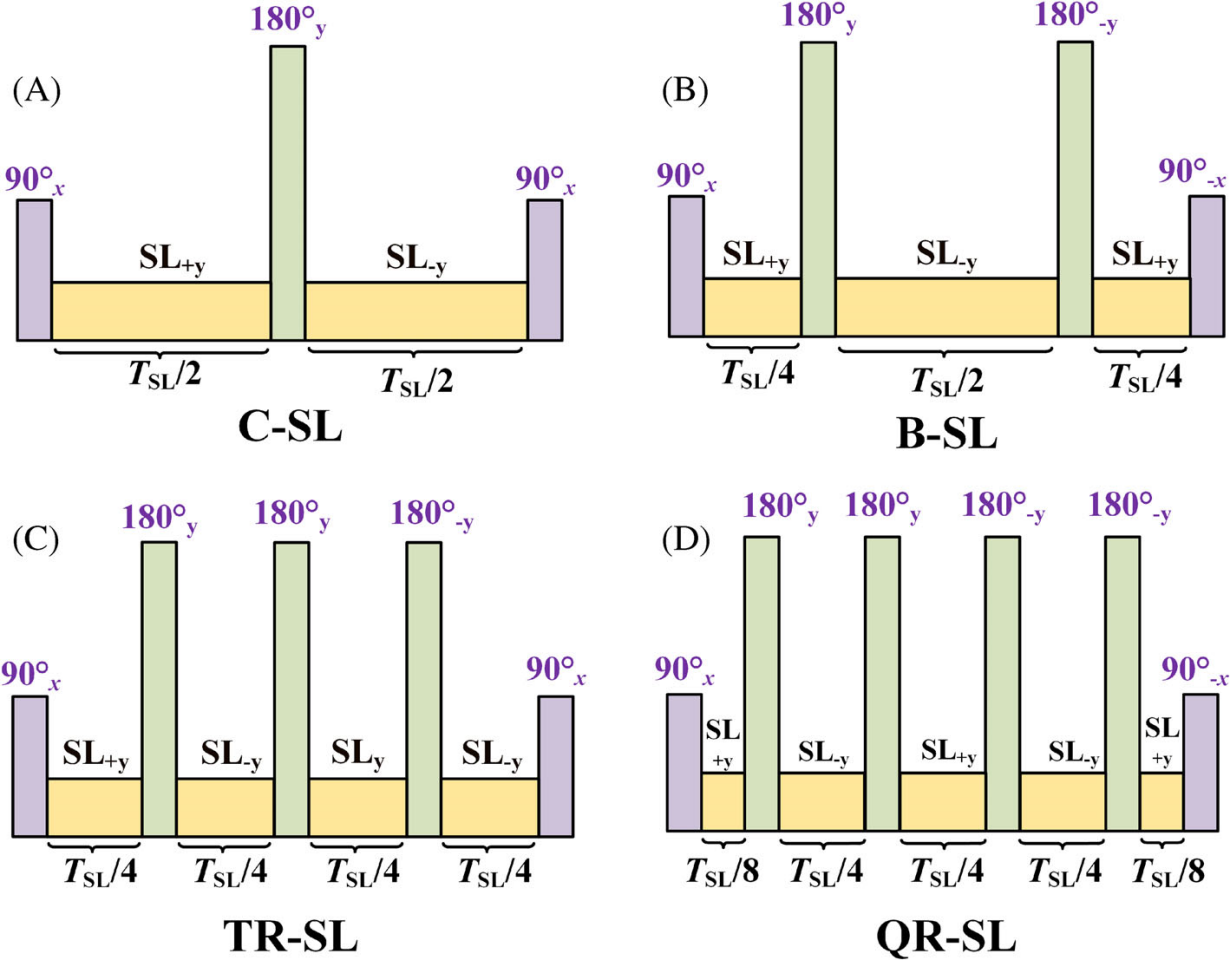
Schematic representations of the spin‐locking (SL) preparation modules explored in this study: composite‐SL (C‐SL^16^) (A); balanced‐SL (B‐SL^18^) (B); triple‐refocused‐SL (TR‐SL^19^) (C); and our proposed quadruple‐refocused‐SL (QR‐SL) (D). The QR‐SL module consists of five SL pulses with phase cycling according to the rotary‐echo principle and four 180° refocusing pulses with opposite phases (+y/+y/−y/−y).

In this study, we compare our proposed QR‐SL module with the C‐SL module proposed by Witschey et al.,^16^ the B‐SL module introduced by Gram et al.,^18^ and the TR‐SL module developed by Pala et al.^19^ The respective sequence diagrams are shown in Figure 3. The QR‐SL module extends the TR‐SL module by incorporating a 180° refocusing pulse to further mitigate the effects of the B_0_ field inhomogeneity. Furthermore, based on the rotary‐echo principle,^14^, ^26^ paired ($180_{y}^{{}^{\circ}}$ and $180_{-y}^{{}^{\circ}}$) RF pulses can balance the refocusing issues caused by the B_1_‐field imperfections.^18^ The SL period is segmented into five pulses with alternating phases throughout the module. We hypothesize that pulses with alternating phases provide enhanced compensation for B_0_ and B_1_ field inhomogeneities.

### 3.2 Analytical comparison

Magnetization dynamics during spin‐lock preparation can be described analytically using matrix propagations as shown in previous work.^16^, ^18^ The QR‐SL module was compared with the proposed C‐SL, B‐SL, and TR‐SL modules by analyzing the magnetization trajectories during the SL preparation process with propagation matrix ***B***. The calculations use rotation (Eq. [[6]) and spin relaxation (Eq. [[7]) matrices. The effective magnetization achieved through these SL modules can be expressed in terms of the appropriate concatenation of matrix–vector multiplications involving the three matrices, ***R***_***x***_, ***R***_***y***_, and ***E***_***ρ***_, as shown in Eqs. (10) and (11). Field inhomogeneity is accounted for by perturbations of the angles *α* (90° pulse), *β* (180° refocusing pulse), and *θ* (tilt of the effective SL field ***B***_***e***_). The specific equations are expressed as follows:

(8) $$R_{90}\left(\alpha \right)=R_{x}\left(\alpha \right),$$

(9) $$R_{180}\left(\beta \right)=R_{y}\left(\beta \right),$$

(10) $${\mathrm{SL}}_{+}\left(\tau \right)=R_{x}\left(-\theta \right)\cdot E_{\rho }\left(\tau \right)\cdot R_{y}\left(\omega_{\mathrm{SL}}\tau \right)\cdot R_{x}\left(\theta \right),$$

(11) $${\mathrm{SL}}_{-}\left(\tau \right)=R_{x}\left(\theta -\pi \right)\cdot E_{\rho }\left(\tau \right)\cdot R_{y}\left(\omega_{\mathrm{SL}}\tau \right)\cdot R_{x}\left(\pi -\theta \right).$$

where ***SL***_+_(τ) and ***SL***_−_(τ) denote an SL pulse with the forward (+*y*) and opposite (−*y*) phases, respectively. The off‐resonance effect of SL, as shown in Figure 2, is addressed through a transformation to the tilted rotating frame in this study. This transformation is specifically represented as the rotation matrix ***R***_***x***_(*θ*) of the x‐axis. The propagation matrix ***B*** for each SL preparation module, shown in Figure 3, is calculated as follows:

(12) $$\begin{array}{cc} B_{C-\mathrm{SL}} & =R_{90}\left(\alpha \right)\cdot {\mathrm{SL}}_{-}\left(\tau /2\right)\cdot R_{180}\left(\beta \right)\\ & \;\times {\mathrm{SL}}_{+}\left(\tau /2\right)\cdot R_{90}\left(\alpha \right) \end{array}$$

(13) $$\begin{array}{cc} B_{B-\mathrm{SL}} & =R_{90}\left(-\alpha \right)\cdot {\mathrm{SL}}_{+}\left(\tau /4\right)\cdot R_{180}\left(-\beta \right)\cdot {\mathrm{SL}}_{-}\left(\tau /2\right)\\ & \;\times R_{180}\left(\beta \right)\cdot {\mathrm{SL}}_{+}\left(\tau /4\right)\cdot R_{90}\left(\alpha \right) \end{array}$$

(14) $$\begin{array}{c} B_{\mathrm{TR}-\mathrm{SL}}=R_{90}\left(\alpha \right)\cdot {\mathrm{SL}}_{-}\left(\tau /4\right)\cdot R_{180}\left(-\beta \right)\cdot {\mathrm{SL}}_{+}(\tau /4)\;\\ \;R_{180}\left(\beta \right)\cdot {\mathrm{SL}}_{-}\left(\tau /4\right)\cdot R_{180}\left(\beta \right)\cdot {\mathrm{SL}}_{+}\left(\tau /4\right)\cdot R_{90}\left(\alpha \right) \end{array}$$

(15) $$\begin{array}{cc} B_{\mathrm{QR}-\mathrm{SL}} & =R_{90}\left(-\alpha \right)\cdot {\mathrm{SL}}_{+}\left(\tau /8\right)\cdot R_{180}\left(-\beta \right)\cdot {\mathrm{SL}}_{-}\left(\tau /4\right)\\ & \;R_{180}\left(-\beta \right)\cdot {\mathrm{SL}}_{+}\left(\tau /4\right)\cdot R_{180}\left(\beta \right)\cdot {\mathrm{SL}}_{-}\left(\tau /4\right)\\ & \;R_{180}\left(\beta \right)\cdot {\mathrm{SL}}_{+}\left(\tau /8\right)\cdot R_{90}\left(\alpha \right) \end{array}$$

By calculating the matrix product of the propagation matrix ***B*** and the ground state magnetization ***M*** = (0, 0, 1)^*T*^, the magnetization *M*_z_ at the end of the SL module was obtained. The solution functions were calculated using *Mathematica* 11.3 (Wolfram Research Inc., Champaign, IL, USA).

### 3.3 Numerical simulations

We obtained the matrix product of the propagation matrix ***B*** and the ground state magnetization ***M*** for each SL module. This *MATLAB*‐based computation (R2020a; MathWorks Inc., Natick, MA, USA) was performed to simulate the effect of inhomogeneities of both fields on the quantitative accuracy of T_1ρ_. The simulation range of B_0_ field inhomogeneity was ±600 Hz, which was achieved by varying the angle (*θ*) in Eq. (10). The range of B_1_ field inhomogeneity was ±50%, which was obtained by adjusting *α* and *β* values in Eqs. (8) and (9). The 100 × 100 cases were used to quantify the range of these inhomogeneities. The matrix ***B*** was used to compute the magnetization vectors *M*_z_ at various *T*_SL_ points, with continuously equal intervals within the range of 0–2 T_1ρ_. In simulations, the susceptibility of banding artifacts was assessed by calculating the residual sum of squares (RSS) between this continuously sampled trajectory and a mono‐exponential fit. In practice, continuous sampling points are rarely used. Consequently, the number of *T*_SL_ points was reduced in simulations to minimize the discrepancy with actual quantitative experiments. Eight points were randomly and uniformly assigned to fit T_1ρ_ to ensure adequate sampling. For comparisons, the same random points were used for each SL module. The quantization error for a single random simulation was denoted as Δ*q*. Simulations were repeated 100 times to obtain the average quantization error Δ*Q*, as follows:

(16) $$\Delta q=\frac{T_{1\rho ,\mathrm{fit}}}{T_{1\rho ,\text{true}}}-1,$$

(17) $$\Delta Q=\frac{1}{100}\sum_{i=1}^{100}\left|\Delta q_{i}\right|.$$

where T_1ρ,fit_ denotes the fitted T_1ρ_ value for a single random simulation, and T_1ρ,true_ denotes the reference T_1ρ_ value.

To evaluate the overall performance of each SL preparation module under varying conditions, repeated simulations were conducted at various *f*_SL_ points (100–4000 Hz) and across different T_1ρ_: T_2ρ_ ratios (1:5 – 5:1). The resulting Δ*Q* and RSS values were analyzed to assess the T_1ρ_ quantification error.

### 3.4 Experimental validation

Five agarose gel phantoms were prepared by heating agarose powder dissolved in water in various concentrations (weight ratios: 2%, 3%, 4%, 5%, and 6%) at elevated temperatures, followed by natural cooling to form a gel. These agarose gels were placed into tubes with a diameter of 20 mm, ultimately resulting in the formation of the phantoms. Additionally, quantitative in vivo experimental validation was performed on articular cartilage of a healthy volunteer. The experimental validation was performed on a Siemens PrismaFit 3T system (Siemens Healthineers AG, Forchheim, Germany) with a knee RF coil (local transmit, 15‐channel receive). All T_1ρ_ preparation modules (Figure 3) were implemented using the open‐source sequence framework, *Pulseq*.^27^

The T_1ρ_ preparation sequence consists of RF pulses, SL pulses, and a gradient crusher, followed by a two‐dimensional turbo spin echo (TSE) imaging sequence. The duration of the 90° excitation and 180° refocusing pulses is 1 ms. For phantom measurements, the T_1ρ_ preparation modules are tested with multiple SL frequencies: *f*_SL_ = 100, 500, and 1000 Hz. T_1ρ_ measurements use a consistent finite number of *T*_SL_ with a sampling scheme of reproducibility‐guided random sampling^28^: *T*_SL_ = 4, 38, 40, 42, and 44 ms. The parameters of the TSE sequence are as follows: field of view (FOV) = 120 × 120 mm^2^, matrix size = 200 × 200, slice thickness = 4 mm, effective echo time (TE) = 24 ms, repetition time = 3000 ms, and echo train length = 4. The duration of each scan is 2: 35 min. For in vivo measurements, the T_1ρ_ preparation modules use an SL frequency of 100 Hz. *T*_SL_ = 4, 20, 40, 60, and 80 ms. The TSE sequence parameters are as follows: FOV = 160 × 160 mm^2^, matrix size = 256 × 256, slice thickness = 4 mm, effective TE = 39 ms, repetition time = 3000 ms, and echo train length = 8. The duration of each scan is 1: 40 min. A fat‐suppression module is used for fat saturation.

Before the T_1ρ_ quantitative measurements, we obtained the inhomogeneity of B_0_ field after the standard shimming procedure and B_1_ field by B_0_ and B_1_ mapping. The B_0_ map was calculated from two images acquired at two different TEs (TE1 = 2.5 ms, TE2 = 3.5 ms).^29^ The B_1_ map was computed based on the double‐angle method (FA1 = 60°, FA2 = 120°).^30^ We used the T_1ρ_ quantitative results obtained under the B_0_ field distribution resulting from the standard shimming procedure as a reference. Then, B_0_ and B_1_ field inhomogeneities were manually introduced to compare their effects on T_1ρ_ quantification across different preparation modules. Setting incorrect flip angles (FAs; ± 20%) for the 90° excitation and 180° refocusing pulses in the preparation module leads to artificial B_1_ field imperfections. Additionally, increasing the gradient in the x‐plane or y‐plane during the SL pulses (e.g., a 20‐cm‐diameter phantom with an x‐plane gradient of 9.6 Hz/cm yields an inhomogeneity of 1.5 ppm) to simulate B_0_ field imperfections. The T_1ρ_ mapping results obtained with manually induced B_0_‐field or B_1_‐field imperfections were compared with those obtained after the standard shimming procedure. This comparison generated a residual map, and the RSS was calculated to evaluate the robustness of different preparation modules to B_0_ and B_1_ field imperfections.

### 3.5 Ethical approval

This study was conducted on a healthy volunteer and was approved by the institutional review board.

## 4 RESULTS

### 4.1 Analytical comparison

The parsed representation of *M*_z_ for the four SL modules described previously was quite complex. Therefore, analytical comparisons were made in specific cases, such as when there is only B_1_‐field inhomogeneity (Δ*ω*_0_ = 0, *θ* = 0) or only B_0_ inhomogeneity (*α* = π/2, *β* = π). The results are presented in the following equations:

(18) $$\begin{array}{c} C-{\mathrm{SL}}_{\theta =0}:\;\\ M_{z}=-e^{-T_{\mathrm{SL}}/T_{1\rho }}\sin{\left(\alpha \right)}^{2}+e^{-T_{\mathrm{SL}}/T_{2\rho }}\cos{\left(\alpha \right)}^{2}\cos\left(\beta \right) \end{array}$$

(19) $$\begin{array}{c} B-{\mathrm{SL}}_{\theta =0}:\;\\ M_{z}=e^{-T_{\mathrm{SL}}/T_{1\rho }}\sin{\left(\alpha \right)}^{2}+e^{-T_{\mathrm{SL}}/T_{2\rho }}\cos{\left(\alpha \right)}^{2} \end{array}$$

(20) $$\begin{array}{c} \mathrm{TR}-{\mathrm{SL}}_{\theta =0}:\;\\ M_{z}=-e^{-T_{\mathrm{SL}}/T_{1\rho }}\sin{\left(\alpha \right)}^{2}+e^{-T_{\mathrm{SL}}/T_{2\rho }}\cos{\left(\alpha \right)}^{2}\cos\left(\beta \right) \end{array}$$

(21) $$\begin{array}{c} \mathrm{QR}-{\mathrm{SL}}_{\theta =0}:\;\\ M_{z}=e^{-T_{\mathrm{SL}}/T_{1\rho }}\sin{\left(\alpha \right)}^{2}+e^{-T_{\mathrm{SL}}/T_{2\rho }}\cos{\left(\alpha \right)}^{2} \end{array}$$

(22) $$\begin{array}{c} C-{\mathrm{SL}}_{\alpha =\pi /2}:\;\\ M_{z}=-e^{-T_{\mathrm{SL}}/T_{1\rho }}\cos{\left(\theta \right)}^{2}-e^{-T_{\mathrm{SL}}/T_{2\rho }}\sin{\left(\theta \right)}^{2} \end{array}$$

(23) $$\begin{array}{c} B-{\mathrm{SL}}_{\alpha =\pi /2}:\;\\ M_{z}=e^{-T_{\mathrm{SL}}/T_{1\rho }}\cos{\left(\theta \right)}^{2}+e^{-T_{\mathrm{SL}}/T_{2\rho }}\sin{\left(\theta \right)}^{2} \end{array}$$

(24) $$\begin{array}{c} \mathrm{TR}-{\mathrm{SL}}_{\alpha =\pi /2}:\;\\ M_{z}=-e^{-T_{\mathrm{SL}}/T_{1\rho }}\cos{\left(\theta \right)}^{2}-e^{-T_{\mathrm{SL}}/T_{2\rho }}\sin{\left(\theta \right)}^{2} \end{array}$$

(25) $$\begin{array}{c} \mathrm{QR}-{\mathrm{SL}}_{\alpha =\pi /2}:\;\\ M_{z}=e^{-T_{\mathrm{SL}}/T_{1\rho }}\cos{\left(\theta \right)}^{2}+e^{-T_{\mathrm{SL}}/T_{2\rho }}\sin{\left(\theta \right)}^{2} \end{array}$$

Equations ((18), (19), (20), (21)) show that the magnetization produced by C‐SL and TR‐SL modules depends on the accuracy of FA *β* of the refocusing pulse when only B_1_ field inhomogeneity is considered. In the B‐SL and the new QR‐SL modules, this effect was not observed due to the use of opposite phase refocusing pulse pairs. In the case of only B_0_ inhomogeneity (Eqs. [(22), (23), (24), (25)]), the magnetization *M*_z_ of the C‐SL and TR‐SL modules is opposite to that of the B‐SL and QR‐SL modules, as the final 90° RF pulse is applied along the x‐axis. However, after applying the absolute value operation, all four modules show identical and optimal behavior. In the case of only B_1_ deviation, the QR‐SL and B‐SL modules perform better.

### 4.2 Numerical simulations

**FIGURE 4:**
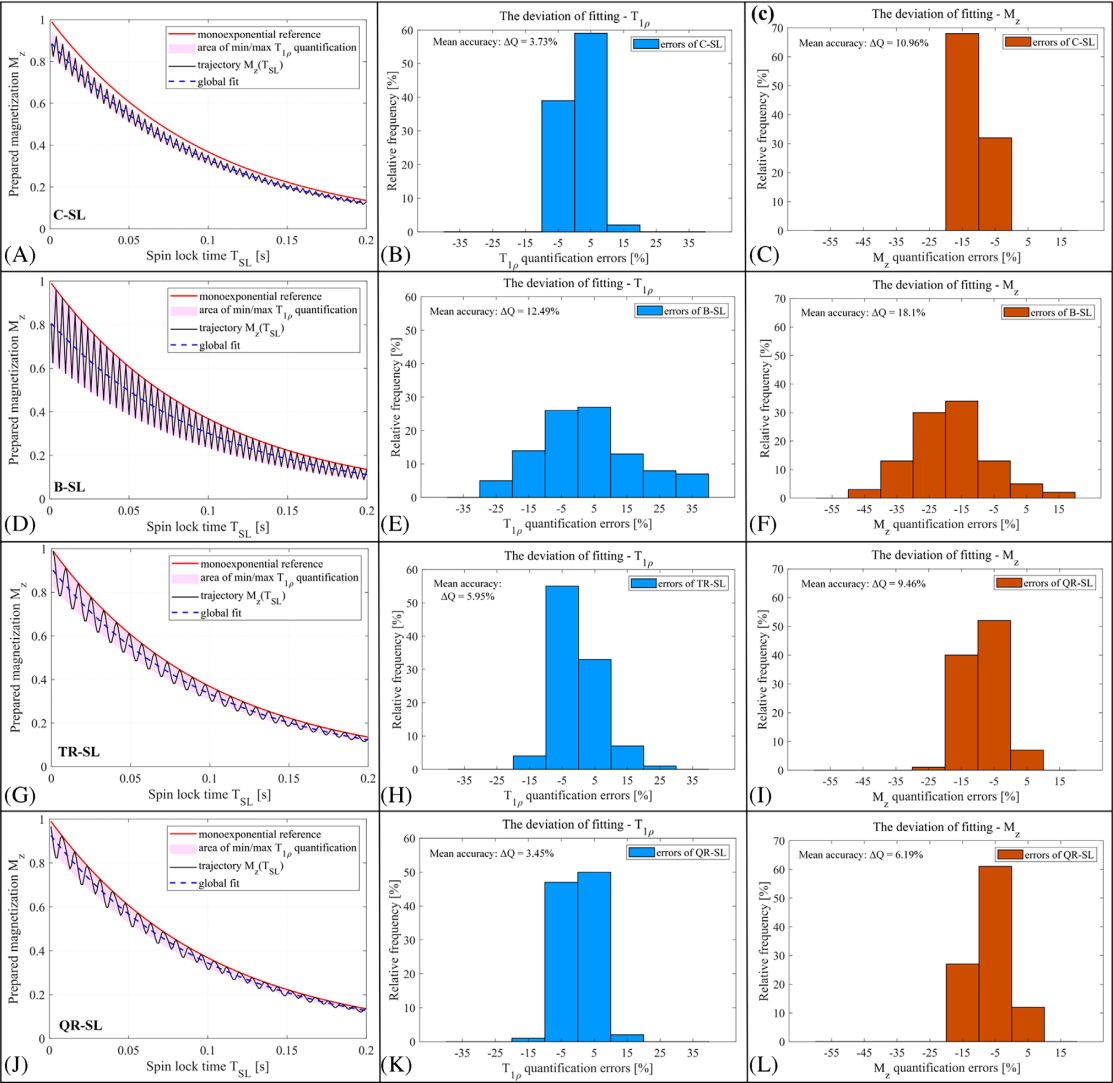
Numerical simulation results. The calculated *M*_z_ trajectories (*black line*) of the composite‐SL (C‐SL) (A), the balanced‐SL (B‐SL) (D), the triple‐refocused‐SL (TR‐SL) (G), and the quadruple‐refocused‐SL (QR‐SL) (J) modules are presented for *f*_SL_ = 500 Hz and field imperfections ∆B_0_ = 350 Hz and ∆B_1_ = −25%. The red line shows the mono‐exponential reference trajectory. The blue dashed line indicates the mono‐exponential fit of the calculated *M*_z_ trajectory. The pink area highlights the quantitative error region. The fitting deviations (Δ*Q*) of T_1ρ_ and *M*_z_ are shown for the C‐SL (B,C), the B‐SL (E,F), the TR‐SL (H,I) and the QR‐SL (J,L) modules.

Figure 4 presents the simulation results of four SL modules with *f*_SL_ = 500 Hz, field imperfections ∆B_0_ = 350 Hz, and ∆B_1_ = −25%. Among these modules, the QR‐SL module displays the lowest oscillation level (RSS) and the lowest mean quantization error (Δ*Q*). In 100‐times repeated simulations, the mean quantization errors of T_1ρ_ of C‐SL, B‐SL, TR‐SL, and QR‐SL modules are 3.73%, 12.49%, 5.95%, and 3.49%, respectively. The mean quantization errors of *M*_z_ of these modules are 10.96%, 18.1%, 9.46%, and 6.19%, respectively, suggesting that the QR‐SL module provides the best fitting accuracy among the tested modules. Notably, although the amplitude of oscillations for C‐SL and QR‐SL modules is comparable, the former shows an additional systematic offset toward lower *M*_z_.

**FIGURE 5:**
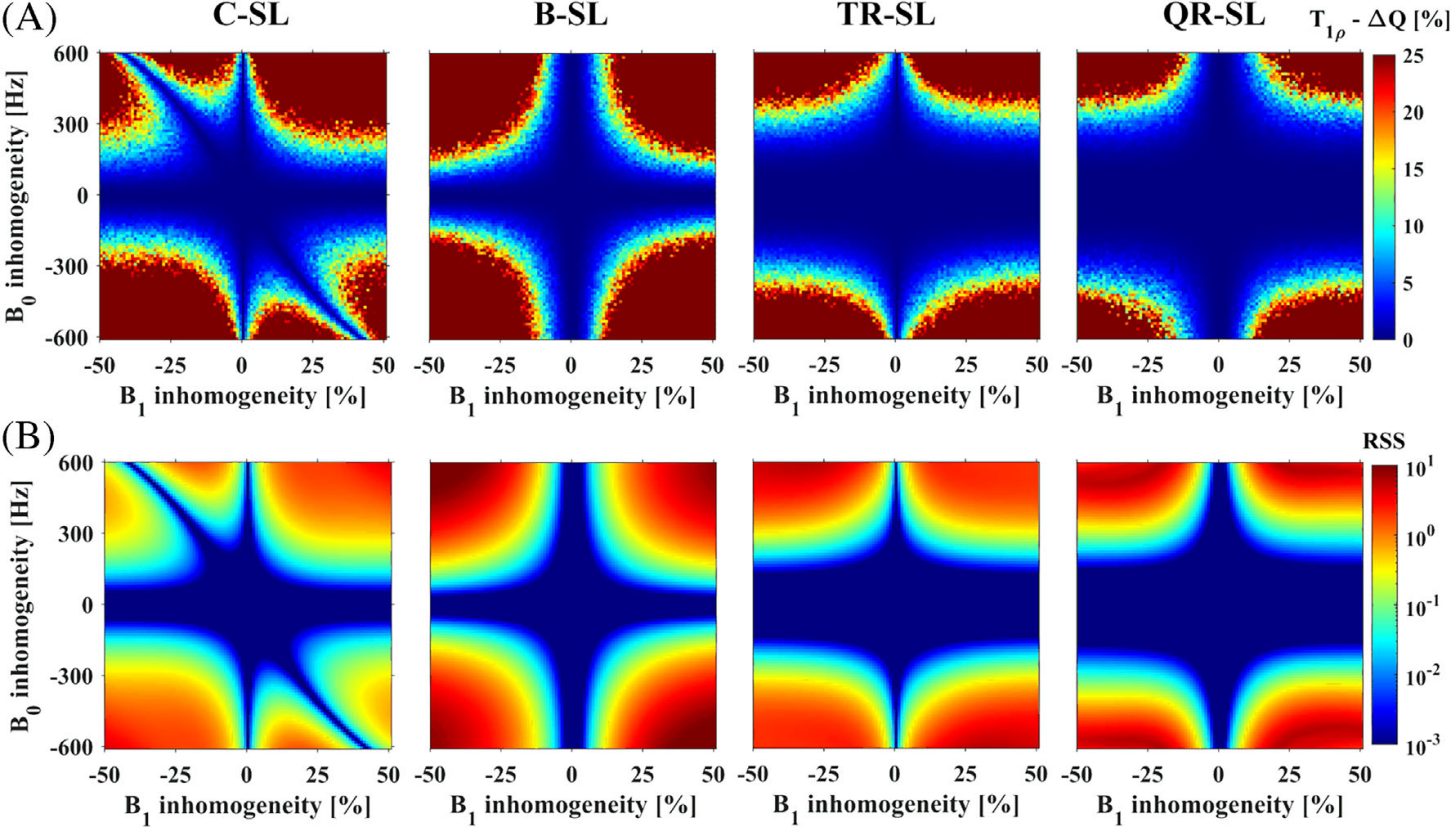
Numerical Bloch simulation comparison of four spin‐locking (SL) modules with *f*_SL_ = 500 Hz and T_1ρ_: T_2ρ_ = 1, and field imperfections ∆B_0_ = ±600 Hz and ∆B_1_ = ±50%. (A,B) The T_1ρ_ quantification error ∆*Q* (A) and the residual sum of squares (RSS) (B) were calculated across the ranges of B_0_ and B_1_ field inhomogeneities with 100 × 100 steps. B‐SL, balanced‐SL; C‐SL, composite‐SL; QR‐SL, quadruple‐refocused‐SL; RSS, residual sum of squares; TR‐SL, triple‐refocused‐SL.

**FIGURE 6:**
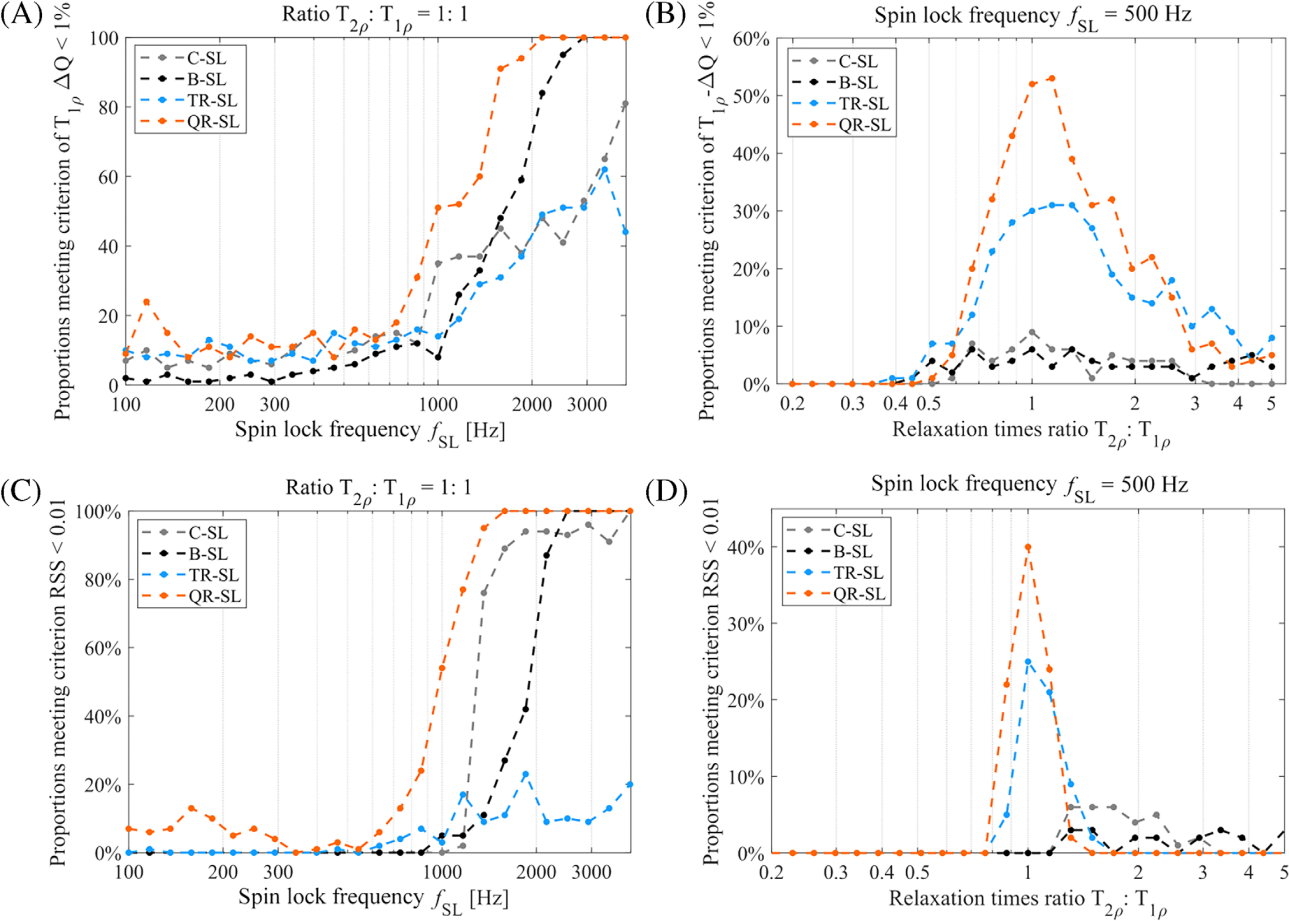
(A,B) The proportions that satisfy the criterion ∆*Q* < 1% are shown for various *f*_SL_ points with T_1ρ_: T_2ρ_ = 1 (A) and various T_1ρ_: T_2ρ_ ratios with *f*_SL_ = 500 Hz (B). (C,D) The proportions meeting the residual sum of squares (RSS) < 0.01 criterion are presented for various *f*_SL_ points with T_1ρ_: T_2ρ_ = 1 (C) and various T_1ρ_: T_2ρ_ ratios with *f*_SL_ = 500 Hz (D). B‐SL, balanced‐SL; C‐SL, composite‐SL; QR‐SL, quadruple‐refocused‐SL; RSS, residual sum of squares; TR‐SL, triple‐refocused‐SL.

Figure 5 presents the mean quantization errors (∆*Q*) and RSS calculated across a 100 × 100 grid of varying B_0_ and B_1_ field inhomogeneities. The C‐SL and TR‐SL modules are less effective than the B‐SL and QR‐SL modules in handing B_1_ field imperfections, as the former remain sensitive to imperfections of the 180° refocusing pulse. The QR‐SL module demonstrates better tolerance to B_0_‐field imperfections than the B‐SL module and similar robustness against B_1_ deviations. The findings from analyses with varying *f*_SL_ are shown in Figure 6A,C, revealing that as *f*_SL_ values increase, the probability of RSS is less than 0.01, and quantization accuracy ∆*Q* improves across all modules. Figure 6B,D presents the analysis of the effect of relaxation‐time ratios. As shown, optimal performance is achieved at T_1ρ_: T_2ρ_ ≈ 1. When comparing the overall performance of each module, the QR‐SL module clearly delivers superior results for both metrics across a wide range of B_0_ and B_1_ field deviations and relaxation‐time ratios.

### 4.3 Experimental validation

**FIGURE 7:**
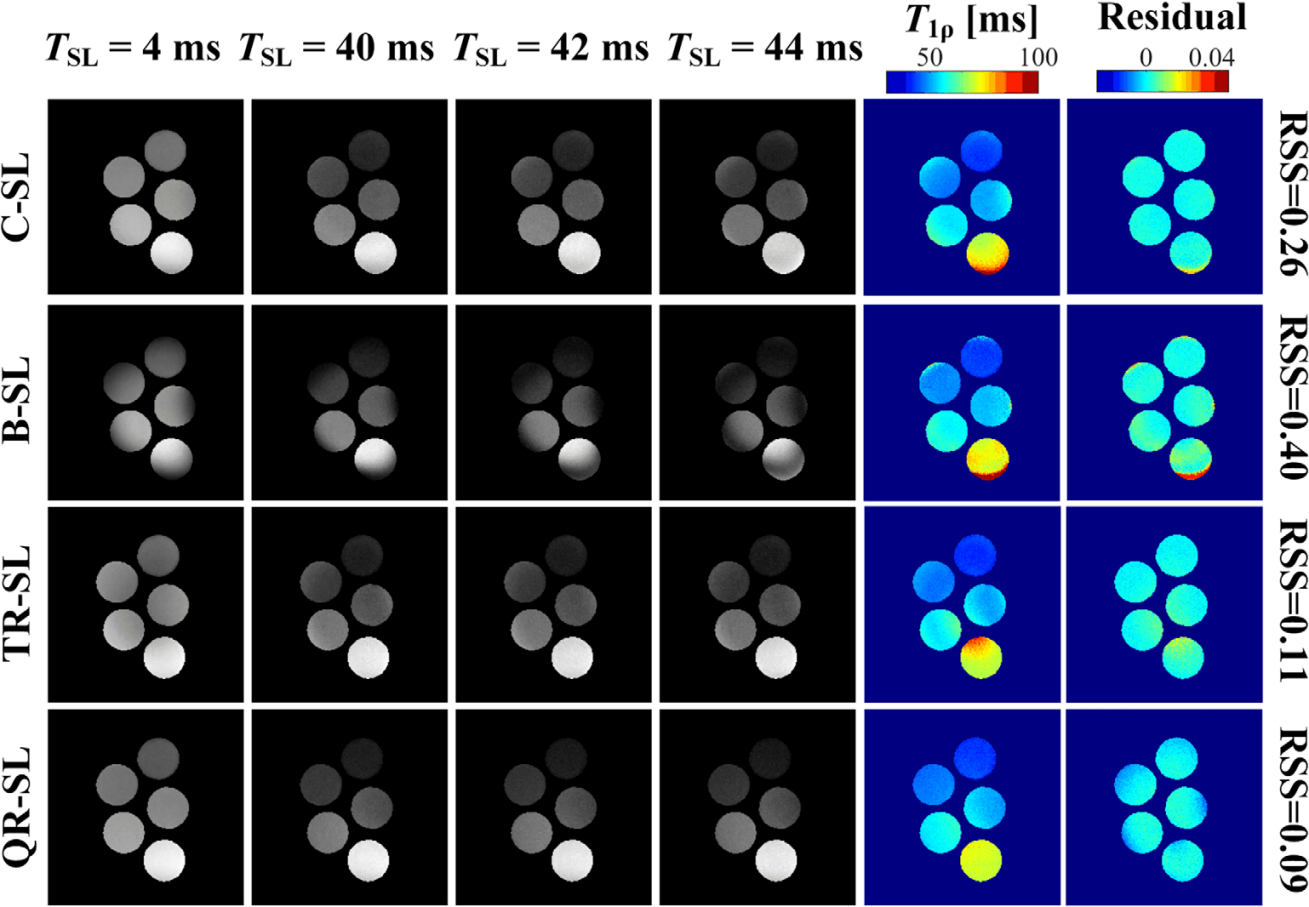
Experimental results based on the agarose gel phantoms. Various T_1ρ_‐weighted images (*T*_SL_ = 4, 40, 42, and 44 ms) for each spin‐locking (SL) module are shown, along with the corresponding T_1ρ_, residual maps, and residual sum of squares (RSS) in the case of *f*_SL_ = 100 Hz and B_1_ field deviation = −20%. The results show the performance of the modules without additional gradient‐induced B_0_ field deviations. B‐SL, balanced‐SL; C‐SL, composite‐SL; QR‐SL, quadruple‐refocused‐SL; TR‐SL, triple‐refocused‐SL.

**FIGURE 8:**
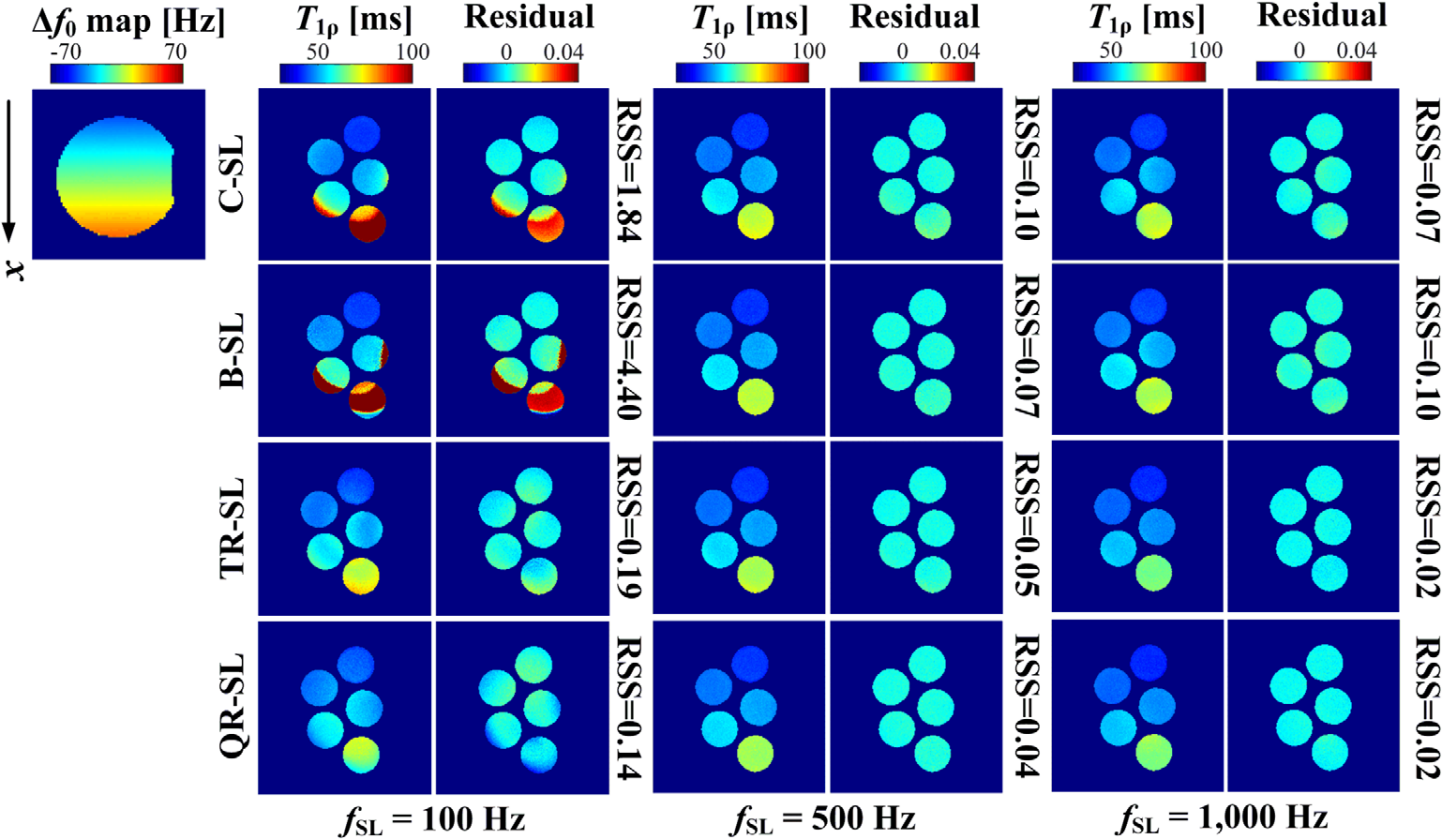
Experimental results based on the agarose gel phantoms for gradient‐induced B_0_ field deviations. For each spin‐locking (SL) module, the corresponding T_1ρ_, residual maps, and residual sum of squares (RSS) are presented for *f*_SL_ = 100, 500, and 1000 Hz, with B_1_ field deviation = −20%, and local field of view varying B_0_ = −58…58 Hz (Δ*f*_0_ map induced by the gradient obtained from a liquid phantom). As *f*_SL_ increases, the performance of each module is significantly enhanced.

**FIGURE 9:**
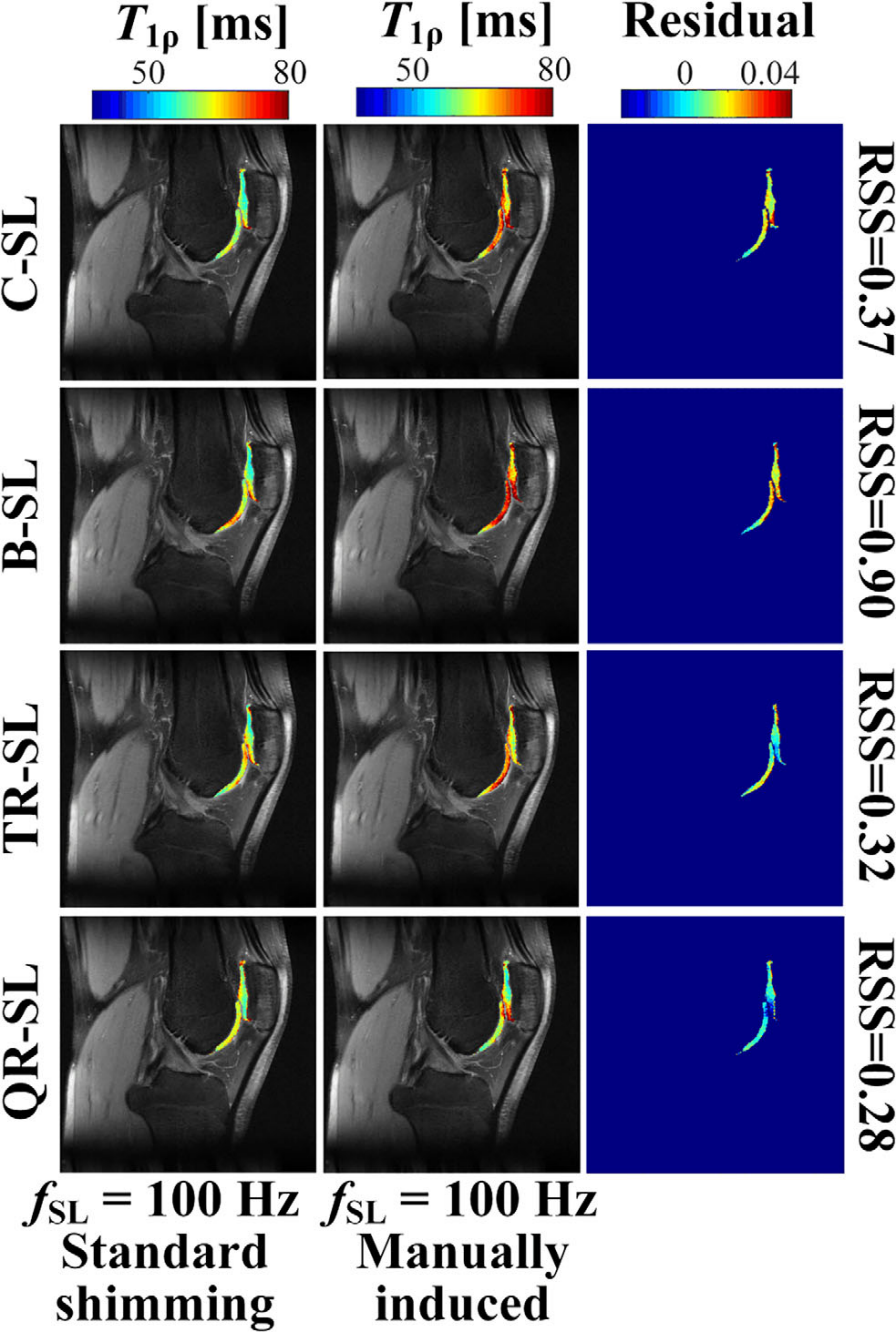
Experimental results based on the knee cartilage for gradient‐induced B_0_‐field deviations. For each spin‐locking (SL) module, the T_1ρ_ under standard shimming, T_1ρ_ under manually induced field imperfections (when *f*_SL_ = 100 Hz, B_1_ field deviation = −20% and local field of view varying B_0_ = −77…77 Hz), residual maps, and residual sum of squares (RSS) are shown. The RSS of the quadruple‐refocused‐SL (QR‐SL) module is decreased by 24.3%, 68.9%, and 12.5%, respectively, compared with the composite‐SL (C‐SL), balanced‐SL (B‐SL), and triple‐refocused‐SL (TR‐SL) modules.

Four SL modules were used to obtain quantitative measurements of T_1ρ_ in the agarose gel phantoms at different *f*_SL_ frequencies (100, 500, and 1000 Hz). Table S1 lists the T_1ρ_ values for the agarose gel samples with various concentrations with *f*_SL_ = 500 Hz. In the absence of manually introduced field inhomogeneities, after the standard shimming procedure, the quantitative results across all modules show remarkable consistency, with a maximum deviation of approximately 2.6%. Figure 7 shows the T_1ρ_‐weighted images, T_1ρ_ maps, and residual maps on the agarose gel phantoms, obtained using the four SL modules with *f*_SL_ = 100 Hz. This experiment was performed with B_1_ field deviation = −20% and B_0_ field after the standard shimming procedure. Under these conditions, the B‐SL module shows more severe artifacts, whereas the TR‐SL and QR‐SL modules display more homogeneous T_1ρ_ maps. The RSS obtained from the C‐SL, B‐SL, TR‐SL, and our proposed QR‐SL are 0.26, 0.40, 0.11, and 0.09, respectively. The RSS of the QR‐SL module decreases by 18.2% compared with the TR‐SL module. Figure 8 shows the T_1ρ_ maps and residual maps for imperfect SL pulses and gradient‐induced off‐resonances (the B_1_ field deviation = −20% and locally FOV‐varying B_0_ = −58…58 Hz for *f*_SL_ = 100, 500, and 1000 Hz, respectively). In these experiments, a gradient in x‐direction was added during the SL pulses to introduce spatially varying B_0_ field imperfections. Figure 8 shows that as the *f*_SL_ increases, the quantization error of each SL module significantly improved, which is consistent with the numerical simulation results. As seen in the figure, at *f*_SL_ = 100 Hz, the banding artifacts associated with the C‐SL and B‐SL modules are significantly more severe, significantly surpassing those of the TR‐SL and QR‐SL modules. Then, we obtained T_1ρ_ maps with *f*_SL_ = 100 Hz for the knee cartilage of a healthy volunteer under both manually induced field imperfections and standard shimming procedure conditions, as shown in Figure 9. Similarly, the B‐SL module exhibits more substantial artifacts, whereas the QR‐SL module demonstrates superior performance. In the presence of B_0_ and B_1_ field inhomogeneities, the QR‐SL module demonstrates a reduction in RSS by 24.3%, 68.9%, and 12.5% compared with the C‐SL, B‐SL, and TR‐SL modules, respectively, for in vivo knee cartilage. Among the four modules, QR‐SL shows the highest performance in compensating for manually introduced B_0_ and B_1_ field imperfections. Furthermore, the C‐SL and TR‐SL modules perform better than the B‐SL module.

**FIGURE 10:**
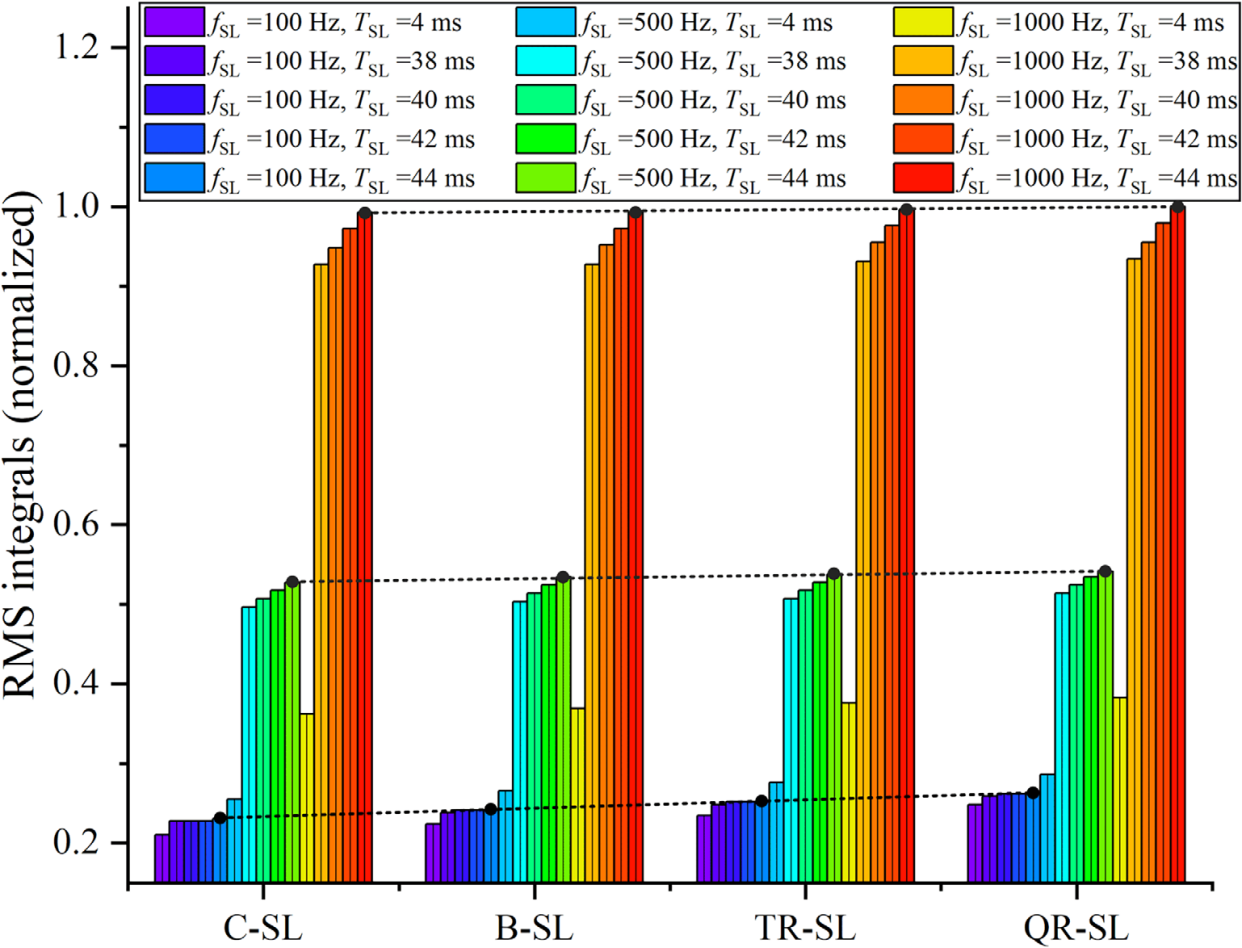
Comparison of root mean square (RMS) radiofrequency integrals for the four T_1ρ_ preparation modules for phantom measurements with *f*_SL_ = 100, 500 and 1000 Hz, and *T*_SL_ = 4, 38, 40, 42, and 44 ms. The RMS integrals of the quadruple‐refocused‐SL (QR‐SL) module show a relative increase of 13.4%, 2.6%, and 0.7% compared with those of the composite‐SL (C‐SL) module when *f*_SL_ = 100, 500, and 1000 Hz and *T*_SL_ = 44 ms, respectively.

In our experiments, we also recorded the root‐mean‐square RF integrals of each SL module for phantom measurements, as shown in Figure 10 (for in vivo measurements, as shown in Figure S1). We observed that RF energy deposition increases for the proposed QR‐SL module, especially at lower *f*_SL_. The root‐mean‐square integrals of the QR‐SL module show a relative increase of 13.4% compared with the C‐SL module with *f*_SL_ = 100 Hz and *T*_SL_ = 44 ms, whereas the increase is only 0.7% when *f*_SL_ = 1000 Hz and *T*_SL_ = 44 ms. These findings suggest that the QR‐SL module remains a viable option compared with the C‐SL module within SAR safety limits.

## 5 DISCUSSION

Based primarily on the research of the B‐SL module,^18^ in this study, we present an extended SL preparation module (QR‐SL) for T_1ρ_ quantification. Our analytical, numerical, and experimental results indicate that the module proposed here outperforms the previous SL preparation modules. The new method provides a more robust compensation for inhomogeneities of both B_0_ and B_1_ fields.

The spin relaxation of SL pulses is a highly intricate process that requires a thorough analysis of the full Bloch equation for the complete description. To simplify this analysis, the relaxation effects during the 90° excitation and 180° refocusing pulses were neglected in the numerical simulations, as well as the influence of the T_1ρ_ dispersion described by spin relaxation theory.^31^ In this study, the magnetization evolution was expressed using propagation matrix ***B***. It is important to note that T_1ρ_ depends on the frequency and off‐resonance of the SL field.^31^ In our simulations, Δ*Q* and RSS were used to represent the experimental accuracy and precision of T_1ρ_ quantification, respectively. Based on Δ*Q* and RSS in the numerical simulations, it can be concluded that QR‐SL exhibits greater robustness against banding artifacts and at the same time results in lower quantization errors. Conversely, the C‐SL and TR‐SL modules have lower B_1_ imperfection compensation abilities. This indicates that pulse schemes with an odd number of refocusing pulses remain sensitive to the imperfections of the 180° pulses. In contrast, the B‐SL and QR‐SL modules use an even number of refocusing pulses, and therefore yield a more robust compensation of B_1_ field imperfections. According to further simulation results presented in Figure S2, increasing the number of 180° pulses (e.g., five, six, or *n*) may exhibit superior performance to the four pulses scheme under specific B_0_ and B_1_ field inhomogeneity conditions. However, an analysis of the global B_0_ and B_1_ field‐inhomogeneity distribution maps shows that the four‐pulse scheme still exhibits superior performance. Nevertheless, this study cannot confirm the existence of a positive correlation between the number of 180° pulses and the robustness of the B_0_ and B_1_ fields. From a scientific rigor perspective, there might be a potential optimization law, but this law needs to be fully elucidated in future studies with extended simulations and experimental setups. We also simulated all six self‐compensating phase combinations of the 180° refocusing pulses in the QR‐SL module, including +y/+y/−y/−y, −y/−y/+y/+y, +y/−y/−y/+y, −y/+y/+y/−y, +y/−y/+y/−y, and −y/+y/−y/+y. Among these, we found that the first four combinations had similar, improved compensation performance, whereas the last two showed degraded performance. In general, it is observed that the T_1ρ_ quantification error tends to be more pronounced at lower *f*_SL_ values. In addition, we also observed asymmetric behavior in the Δ*Q* and RSS heatmaps of the C‐SL module (Figure 5), which differs from the other modules. We speculate that it may be due to the reverse phase of the second 90° pulse or mutual cancellation effects between B_0_ and B_1_ field imperfections. When the effective spin‐lock axis is tilted by a B_0_ deviation, magnetization becomes precisely aligned along this axis because of a B_1_ deviation. In this case, no banding artifacts occur despite the field deviations. In addition, for C‐SL and TR‐SL modules, the T_1ρ_‐prepared magnetization is flipped to the *‐z* axis, whereas for B‐SL and QR‐SL modules, it is flipped to the +*z* axis. This orientation may have an effect on T_1_ contamination due to a slight delay before the signal readout. For these modules, a spin echo–type readout sequence generally outperforms a gradient recalled–echo sequence with a small FA excitation pulse.^19^

The numerical simulations were validated with experimental results, comparing each SL module directly with the same parameters. After the standard shimming procedure of B_0_ field, the four modules exhibited comparable T_1ρ_ values. In scenarios where additional field inhomogeneities were present, the QR‐SL module exhibited superior performance based on analysis of the RSS and T_1ρ_‐weighted images. At *f*_SL_ = 100 Hz, both C‐SL and B‐SL modules showed poor performance. If either C‐SL or B‐SL modules are deemed necessary, it may be advantageous to use them at higher *f*_SL_ values. Meanwhile, this study confirms that the C‐SL module outperforms the B‐SL module. Additionally, the artifacts decreased with increasing SL frequency under the same B_0_ and B_1_ field imperfections. Although our experiments did not guarantee that the QR‐SL module yields optimal results across all scenarios of inhomogeneities of B_0_ and B_1_ field, it has demonstrated superior performance in both numerical simulations and experimental measurements under the investigated conditions.

The present study has several limitations. First, for T_1ρ_ quantitative comparison, this study focused on the preparation modules that have been shown to perform well according to the recent literature.^16^, ^18^, ^19^ Furthermore, the number of experimental samples was limited, and a limited number of *T*_SL_ points^28^ were chosen for the experiments. The selection of different *T*_SL_ values for phantom and in vivo measurements is based primarily on the differences in physical properties and experimental objectives of the two. This differentiated *T*_SL_ selection ensures that the experimental goals of both phantoms and in vivo measurements are optimally achieved. The choice of *T*_SL_ is an important factor that can be addressed by using Cramér–Rao.^32^ The complex magnetization evolution during SL makes T_1ρ_ quantification susceptible to inhomogeneities of both fields and T_2ρ_ effects. Consequently, obtaining an accurate T_1ρ_ value at finite *T*_SL_ points is challenging. We also observed that selecting different *T*_SL_ points not only resulted in differences in the fitted T_1ρ_ values, but also in varying levels of artifacts. Although adiabatic excitation and refocusing pulses could further stabilize the SL preparation modules,^33^ only on‐resonance hard pulse SL modules were investigated here. Due to the limitation of SAR at 3 T, the maximum SL frequency used in the sequence was restricted to *f*_SL_ = 1000 Hz. Combining a constant amplitude technique^21^, ^34^ with the QR‐SL module might enhance performance in cases that are not limited by SAR, such as in low‐field and ultralow‐field MRI. For in vivo quantitative imaging options at high main magnetic fields such as 3 T and above, C‐SL remains perhaps the most viable. Because the four refocusing pulses are incorporated into the new QR‐SL module, SAR limits are likely to constrain the application of the technique. Conversely, the QR‐SL module may be more suitable for low‐field and ultralow‐field MRI applications. Future studies could further investigate the suitability of QR‐SL for improved T_2ρ_ quantification,^35^, ^36^ and a direct comparison of QR‐SL with previously published adiabatic T_1ρ_ methods should be performed.^37^ However, it is important to note that adiabatic techniques have been shown to provide excellent image quality and robustness^38^ but effectively provide T_1ρ_ values depending on the shape of the adiabatic pulse modulation.^39^ Another possible application of the quadruple refocused SL technique might be the detection of ultraweak alternating biomagnetic fields, which requires superb compensation for static field deviations.^40^, ^41^, ^42^, ^43^

## 6 CONCLUSION

In summary, theoretical, numerical, and experimental comparisons of different T_1ρ_ preparation modules reveal that the proposed QR‐SL module has the potential to provide better B_0_ and B_1_ field inhomogeneity compensation. Compared with C‐SL, B‐SL, and TR‐SL modules, the QR‐SL module may be particularly suitable for MR platforms with larger B_0_ and B_1_ field imperfections that are not limited by SAR, such as ultralow‐field MRI systems.

## CONFLICT OF INTEREST

Nothing to report.

## Supporting information


**Figure S1.** Comparison of root mean square (RMS) radiofrequency (RF) integrals for the four T_1ρ_ preparation modules for in vivo measurements with *f*_SL_ = 100 Hz and *T*_SL_ = 4, 20, 40, 60, 80 ms. The RMS integrals of the quadruple‐refocused spin‐locking (QR‐SL) module show a relative increase of 11.8% than those of the composite spin‐locking (C‐SL) module when *f*_SL_ = 100 Hz and *T*_SL_ = 80 ms, respectively.
**Figure S2**. Numerical Bloch simulation comparison of four different 180° pulses with *f*_SL_ = 500 Hz and T_1ρ_: T_2ρ_ = 1, field imperfections ∆B_0_ = ±600 Hz, and ∆B_1_ = ±50%.
**Figure S3**. Numerical Bloch simulation comparison of quadruple‐refocused spin‐locking (QR‐SL) and paired self‐compensated spin‐locking (PSC‐SL) modules with *f*_SL_ = 500 Hz and T_1ρ_: T_2ρ_ = 1, field imperfections ∆B_0_ = ±600 Hz, and ∆B_1_ = ±50%.
**Table S1.** Quantitative T_1ρ_ value (mean ± standard deviation) of the agarose gel phantoms with *f*_SL_ = 500 Hz.

## Data Availability

The sequences and source code related to this study have been made available on Github: https://github.com/CQU‐NMRLab/QR‐SL‐open_data_and_code.
