# Supplementary material for: Quadruple‐refocused spin‐locking: A robust method for high‐amplitude T1ρ imaging
Source: Magn Reson Med. 2025 Jun 28;94(5):2023–37. doi: 10.1002/mrm.30621 (PMC12322236; doi:10.1002/mrm.30621)
Supplement: Supplementary file 1 — Figure S1. Comparison of root mean square (RMS) radiofrequency (RF) integrals for the four T1ρ preparation modules for in vivo measurements with f SL = 100 Hz and T SL = 4, 20, 40, 60, 80 ms. The RMS integrals of the quadruple‐refocused spin‐locking (QR‐SL) module show a relative increase of 11.8% than those of the composite spin‐locking (C‐SL) module when f SL = 100 Hz and T SL = 80 ms, respectively. Figure S2. Numerical Bloch simulation comparison of four different 180° pulses with f SL = 500 Hz and T1ρ: T2ρ = 1, field imperfections ∆B0 = ±600 Hz, and ∆B1 = ±50%. Figure S3. Numerical Bloch simulation comparison of quadruple‐refocused spin‐locking (QR‐SL) and paired self‐compensated spin‐locking (PSC‐SL) modules with f SL = 500 Hz and T1ρ: T2ρ = 1, field imperfections ∆B0 = ±600 Hz, and ∆B1 = ±50%. Table S1. Quantitative T1ρ value (mean ± standard deviation) of the agarose gel phantoms with f SL = 500 Hz. [file MRM-94-2023-s001.docx]

Table S1. Quantitative *T*_1ρ_ value (Mean ± Standard deviation) of the agarose gel phantoms with *f*_SL_ = 500 Hz.

| Concentration  Module | 6% (ms) | 5% (ms) | 4% (ms) | 3% (ms) | 2% (ms) |
| --- | --- | --- | --- | --- | --- |
| C-SL | 20.0 ± 3.1 | 26.1 ± 4.0 | 30.3 ± 4.6 | 35.9 ± 5.4 | 53.7 ± 8.2 |
| B-SL | 20.2 ± 3.1 | 26.0 ± 4.0 | 30.2 ± 4.6 | 35.6 ± 5.4 | 52.9 ± 8.0 |
| TR-SL | 20.1 ± 3.3 | 26.2 ± 4.0 | 30.5 ± 4.7 | 36.0 ± 5.5 | 54.1 ± 8.2 |
| QR-SL | 20.4 ± 3.1 | 26.1 ± 4.0 | 30.2 ± 4.6 | 35.5 ± 5.4 | 52.7 ± 8.0 |


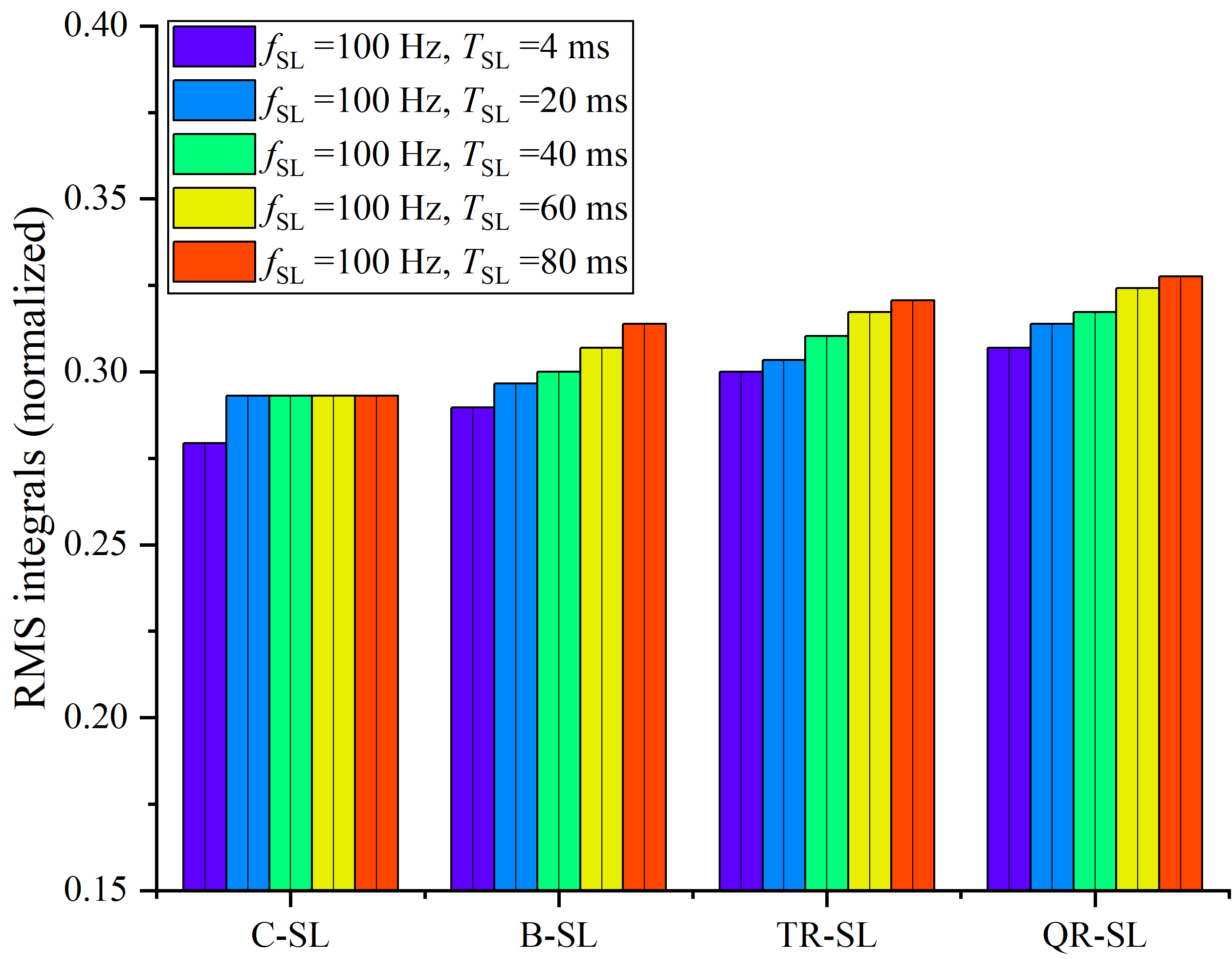


Figure S1. Comparison of root mean square (RMS) RF integrals for the four *T*_1ρ_ preparation modules for in vivo measurements with *f*_SL_=100 Hz, and *T*_SL_ = 4, 20, 40, 60, 80 ms. The RMS integrals of the QR-SL module show a relative increase of 11.8% than those of the C-SL module when *f*_SL_ = 100 Hz and *T*_SL_ = 80 ms, respectively.

For the question of whether five, six or n 180° pulses are preferable to four pulses. According to our further simulation results, increasing the number of 180° pulses (e.g., five, six, or n) may exhibit superior performance to the four pulses scheme under specific B0 and B1 field inhomogeneity conditions. However, an analysis of the B0 and B1 field inhomogeneity distribution maps shows that the four pulses scheme still exhibits superior performance, as shown in Figure S2.


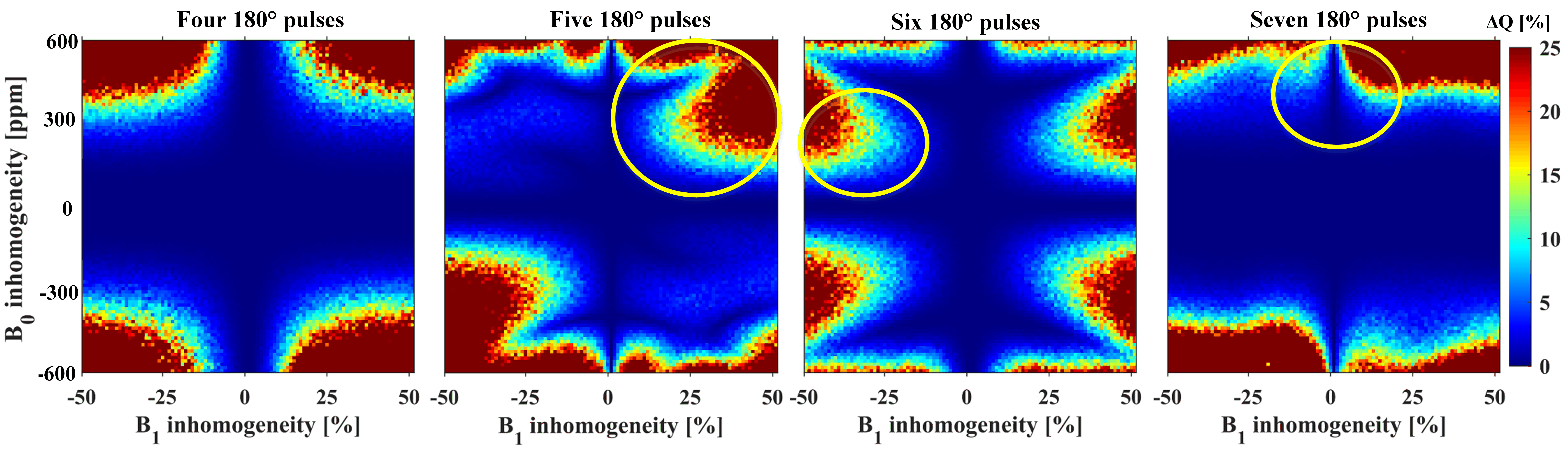


Figure S2. Numerical Bloch simulation comparison of four different 180° pulses with *f*_SL_ = 500 Hz and T_1ρ_: T_2ρ_ = 1, field imperfections ∆B_0_ = ±600 Hz and ∆B_1_ = ±50%.

We simulated PSC-SL here and compared the simulation results with the QR-SL module, as shown in Figure S3.


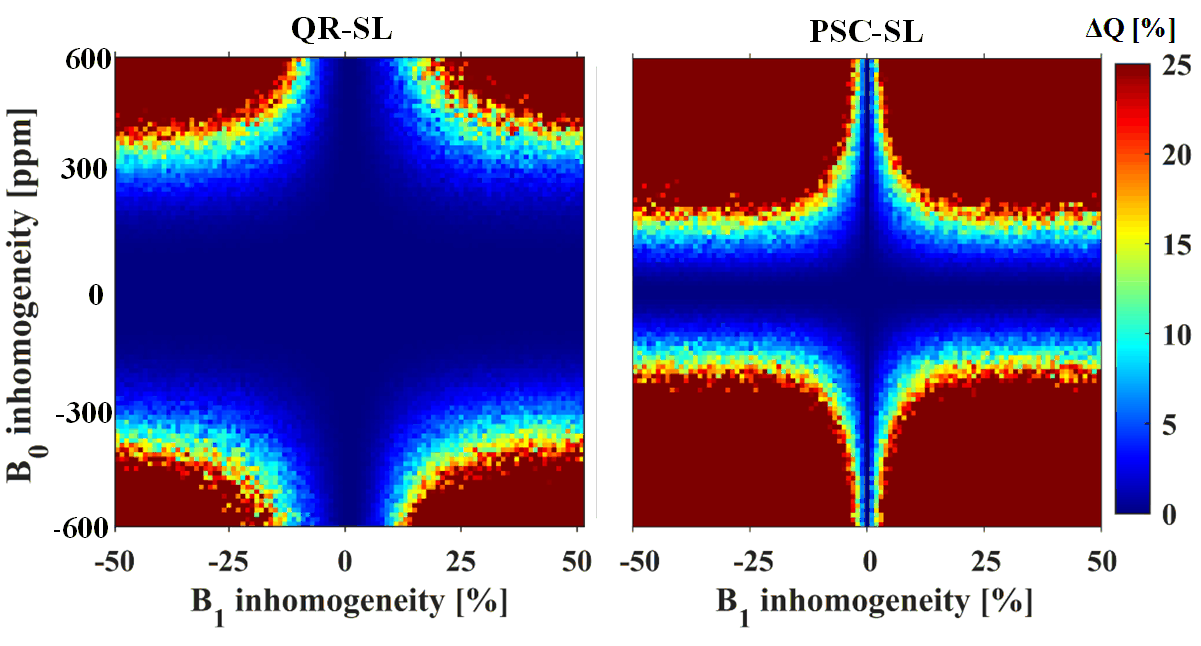


Figure S3. Numerical Bloch simulation comparison of QR-SL and PSC-SL modules with *f*_SL_ = 500 Hz and T_1ρ_: T_2ρ_ = 1, field imperfections ∆B_0_ = ±600 Hz and ∆B_1_ = ±50%
